# Analysis of serum NLR combined with intraoperative margin condition to predict the prognosis of cervical HSIL patients undergoing LEEP surgery

**DOI:** 10.1515/med-2024-1101

**Published:** 2025-03-06

**Authors:** QiaoXian Tian, JiaYao Ma, YaHua Wu, LingYun Wang, WenJiao Yang

**Affiliations:** Department of Obstetrics and Gynecology, Fengcheng Hospital, Shanghai, 201411, China; Department of Obstetrics and Gynecology, Qingpu Branch of Zhongshan Hospital Affiliated to Fudan University, No.1158 Park East Road, Qingpu District, Shanghai, 201700, China; Department of Obstetrics and Gynecology, Fengcheng Hospital, No.9983 Chuannanfeng Road, Fengxian District, Shanghai, 201411, China

**Keywords:** high-grade squamous intraepithelial lesion, NLR, positive margins, recurrence/residual

## Abstract

**Objective:**

This study analyzed the prognosis of patients with high-grade squamous intraepithelial lesion (HSIL) after loop electrosurgical excision procedure (LEEP) and elucidated the predictive value of neutrophil-to-lymphocyte ratio (NLR) and margin condition in relation to prognostic recurrence.

**Methods:**

A total of 209 patients were included, with 46 cases in the recurrence/residual group, 156 cases in the no recurrence/residual group, and 7 cases lost to follow-up. General information, past history and comorbid underlying diseases, laboratory tests, and other relevant clinical information were compared between the two groups. The ROC curves were plotted to assess the diagnostic values of NLR, platelet-to-lymphocyte ratio (PLR), and lymphocytes. Log-rank test was conducted to plot the Kaplan Meier curves to assess the occurrence of recurrence/residual. Risk factors for the occurrence of recurrence/residual in patients during follow-up were analyzed.

**Results:**

Patients with high-risk human papillomavirus (HR-HPV) infection, positive margins, and higher PLR and NLR had a higher risk of recurrence/residual at follow-up. HR-HPV infection, positive margins, and higher levels of PLR and NLR showed significant hazard ratios. High NLR, positive margins, and HR-HPV infection resulted in poor prognosis and the occurrence of recurrence or residual.

**Conclusion:**

NLR levels and positive margins may be markers for predicting HSIL recurrence/residual lesions after LEEP.

## 1 Introduction

High-grade squamous intraepithelial lesion (HSIL) is a precancerous lesion closely associated with the development of cervical squamous cell carcinoma. HSIL may progress to invasive carcinoma if left untreated [1]. The American Society for Colposcopy and Cervical Pathology published the “Risk-Based Management Consensus Guidelines for Abnormal Cervical Cancer Screening Tests and Cancer Precursors (2019),” which recommends prioritizing local excision of HSIL in all cervical intraepithelial neoplasia (CIN) II and III patients [2], including loop electrosurgical excision procedure (LEEP) [3]. This procedure not only effectively removes the lesions, but also preserves the function and anatomy of the cervix. However, there are still some patients with CIN recurrence and residual after LEEP [4,5]. A meta-analysis found that the overall concordance between colposcopic diagnosis and histopathologic findings was only 68.35% [6]. In addition, Perkins et al. [3] showed that the sensitivity of cytologic screening for cervical HSIL was only 53%. Therefore, those with high risk after cytologic screening, including HSIL, should be followed up more intensively or treated aggressively, even if colposcopy fails to detect the abnormality. However, fewer studies have been conducted to further stratify and analyze the factors associated with prognostic recurrence of HSIL, and the optimal treatment plan is still controversial.

Current procedures for the prevention and control of cervical lesions, while improving early detection rates, do not allow the assessment of risks and lesion progression. Neutrophil-to-lymphocyte ratio (NLR) and platelet-to-lymphocyte ratio (PLR) are associated with the diagnosis and prognosis of solid tumors, such as gastric cancer, hepatocellular carcinoma, and colorectal cancer, in some studies [7–10]. NLR, as a reliable indicator for assessing immune-inflammation, and PLR, as a biomarker reflecting inflammatory response and immune function of the human body, are closely associated with malignant tumors. Meanwhile previous studies emphasize the utility of PLR and NLR as non-invasive biomarkers in assessing disease severity, guiding therapeutic decisions, and predicting cervical cancer outcomes [11,12]. The establishment of these associations further strengthens the potential clinical value of PLR and NLR in aiding risk stratification, treatment selection, and patient prognosis. In addition, there are few studies addressing the prediction of prognosis by NLR in patients with HSIL.

Positive margins are usually considered a risk factor for residual/recurrent lesions. A 7-year retrospective study found that positive margins in post-cervical conization specimens were a high risk factor for residual/recurrent cervical lesions [13]. Wang et al. found that cytologic abnormalities (including HSIL), high-risk HPV infection, and multi-quadrant involvement were associated with positive margins and residual lesions [14]. The rate of positive margins is higher in menopausal women than in non-menopausal women [14]. Although positive margins is considered to be a key factor in lesion residue/recurrence after hysterectomy, a negative margin does not imply that the lesion has been completely removed, and the role of margin condition in lesion residue/recurrence and postoperative pathologic grading is controversial.

Based on a retrospective cohort study of HSIL patients, this study focused on investigating NLR and margin condition in HSIL patients and evaluating their relationship with the prognostic features of HSIL, so as to provide a basis for clinical diagnosis, prognostic testing, and treatment planning in HSIL.

## 2 Materials and methods

### 2.1 General information and grouping

A total of 209 patients with HSIL admitted to Fengcheng Hospital from January 2017 to January 2022 were retrospectively analyzed. The patients were categorized into the recurrence/residual group (*n* = 46) and no recurrence/residual group (*n* = 156) according to whether they had recurrence or residual 1 year after surgery.

### 2.2 Inclusion and exclusion criteria

Inclusion criteria included: (1) all were diagnosed with HSIL by pathologic biopsy, including CIN II and III, (2) all underwent cervical cold knife conization, (3) follow-up time was ≥1 year, (4) relevant clinical data were complete, and (5) all underwent cervical surgery for the first time.

Exclusion criteria included: (1) patients with history of cervical surgery, (2) patients with other systemic major diseases, (3) women during pregnancy, and (4) patients with vaginal wall CIN.

### 2.3 Histopathological diagnosis of the cervix

Cervical tissue was taken under colposcopy, and cervical tube scraping was performed when necessary. The specimens were put into special tubes and interpreted sequentially by experienced pathologists in accordance with the 4th edition of the WHO Classification of Tumors of Female Reproductive Organs (2014). The diagnosis was HSIL [15].

### 2.4 General information

General information was collected, including age, pregnancy status, delivery status, body mass index (BMI), and routine preoperative investigations, such as HPV infection and pathologic findings. Blood samples were collected from patients and healthy medical examiners at admission, and blood routine tests were performed. After fasting for at least 12 h, blood biochemical parameters were measured by Beckmann 780, including white blood cell count (WBC), hemoglobin (HGB), platelet count (PLT), mean platelet volume (MPV), neutrophil and lymphocyte counts. NLR was calculated by taking the ratio of absolute neutrophil count to lymphocyte count, and PLR was defined as the absolute platelet count relative to the absolute lymphocyte count.

### 2.5 LEEP

The bladder lithotomy position was taken, and the vulva and vagina were disinfected routinely. Cervical lesions were marked with Lugol iodine solution, and excision was performed 0.5 cm outside the iodine non-colored area. The power of excision can be set at 40–60 W, the power of electrocoagulation at 20–40 W, and the power of excision and coagulation at 35–45 W. The wound hemostasis was performed by thermocoagulation or spherical electrode, and the fresh specimens were sent for examination.

### 2.6 Identification of positive margins

Cervical squamous intraepithelial lesions <1 mm from the margin were defined as margin-positive, including lesions immediately adjacent to or near the margin.

### 2.7 HPV screening

The specimens were collected 3–5  days after menstruation. Cervical secretions were wiped clean with a sterile cotton swab, and then epithelial exfoliated cells at the junction of squamous epithelium and columnar epithelium were extracted with a cervical brush. HPV-DNA was detected using the Roche Cobas 4800 HPV-DNA detection system and the human tumor virus genotyping kit (PCR-reverse dot blot hybridization). The high-risk types were 16, 18, 31, 33, 35, 39, 45, 51, 52, 56, 58, 59, 82, and 68. The time interval of each review was at least half a year. Patients who test positive for the same type of HPV-HPV on more than two consecutive occasions are considered persistent HPV-HPV infection.

### 2.8 Follow-up and determination criteria

Patients were followed up after surgery, with reviews every 3 months for 1 year and every 6 months after 1 year. Of the 202 patients, the shortest follow-up period was 1 year and the longest was 3 years. HPV tests and assessments of HSIL clinical residual were performed at each follow-up visit. Cervical cancer staging was judged according to FIGO 2009 staging criteria. Recurrence refers to negative cytological findings within 6 months of LEEP and pathological confirmation as CIN II or more severe lesion after 6 months. Residual refers to a positive pathologic test within 6 months of LEEP with a diagnosis of CIN II or more severe lesions. For those with abnormal HPV-HC2 and cytology results within the first 6 months of postoperative follow-up, an experienced gynecologist performed colposcopy, vinegar-white test and Lugol’s iodine staining of suspicious lesions, and cervical biopsy and/or endocervical curettage and cervical biopsy. On re-examination, there was no residual if CIN I and chronic inflammation of the cervix were confirmed. Histopathological diagnosis confirmed residuals if there were HSIL (CIN II or III) or higher grade lesions. Recurrence/residual rate = residual rate + recurrence rate. The study endpoint event was defined as recurrence or residual, and the follow-up ended in January 2023. During the follow-up period, seven patients were excluded due to (1) invalid, unsatisfactory, or missing specimens; (2) large amount of missing data; and (3) withdrawal from the study.

### 2.9 Statistical analysis

SPSS 26.0 was used for statistical analysis. Measurements were tested for normality, and those obeying normal distribution were expressed as mean ± standard deviation (SD), and comparisons between two groups were made using the *t*-test. Non-normally distributed data were expressed as median and interquartile spacing M, and comparisons between groups were made using the Wilcoxon rank sum test. The chi-square test was performed for count data. Independent prognostic factors associated with recurrent/residual lesions were determined using univariate/multivariate Cox regression. Hazard ratios (HRs) were calculated. Forest plots of the above regressions were drawn by GraphPad Prism 8.0.2, while Kaplan Meier curves were plotted using the Log rank test. Binary Logistic Regression Analysis was used to evaluate the predictive value of margin positivity combined with NLR. The predicted efficacy was analyzed by receiver operating curve (ROC) analysis, and the area under the curve (AUC), confidence interval, sensitivity, specificity, and cut-off values were obtained. MedCalc software was used to compare whether there was a statistical difference in AUC between the different indicators. A significant difference was defined as *P* < 0.05.


**Informed consent:** All patients gave informed consent.
**Ethical approval:** All procedures performed in this study involving human participants were in accordance with the ethical standards of the institutional and/or national research committee and with the 1964 Helsinki Declaration and its later amendments or comparable ethical standards and has been approved by the Ethics Committee of Fengcheng Hospital (No. 201507FC21).

## 3 Results

### 3.1 General clinical data of patients in recurrence/residual and no recurrence/residual groups

A total of 202 patients were enrolled in this study and divided into two groups: 46 patients in the recurrence/residual group and 156 patients in the no recurrence/residual group. The clinicopathological characteristics of all subjects are shown in Table 1. There was no significant difference in age between the two groups (*P* > 0.05). The age of patients in the recurrence/residual group ranged from 49 to 57 with a mean age of (53.40 ± 4.11) years. The age of patients in the no recurrence/residual group ranged from 49 to 56 with a mean age of (52.80 ± 3.33) years. Apart from this, no significant difference was found in BMI, past medical history, pathological examination, and routine blood tests (HGB, WBC, PLT, MPV, and neutrophil count) between the two groups (*P* > 0.05). The rate of positive high-risk human papillomavirus (HR-HPV), the rate of positive margins, and levels of NLR and PLR were higher, while neutrophil count was lower in the recurrence/residual group than in the no recurrence/residual group (all *P* < 0.05).

**Table 1:** General clinical data of patients in recurrence/residual and no recurrence/residual groups

Characteristics	Recurrence/residual (*n* = 46)	No recurrence/residual (*n* = 156)	*P* value
Age (year)	53.40 ± 4.11	52.80 ± 3.33	0.311
BMI (kg/m2)	25.84 ± 2.68	25.33 ± 3.05	0.301
**Disease history**			
Hyperlipidemia	22 (48%)	77 (49%)	0.328
Hypertension	24 (52%)	69 (44%)	0.512
Diabetes	19 (41%)	81 (52%)	0.331
**Pregnancy**			0.087
Yes	18 (39%)	86 (55%)	
No	28 (61%)	70 (45%)	
**Delivery**			0.641
Yes	21 (46%)	79 (51%)	
No	25 (54%)	77 (49%)	
**Menopause**			0.512
Yes	24 (52%)	80 (51%)	
No	22 (48%)	76 (49%)	
**Gland invasive**			0.384
No	19 (41%)	69 (44%)	
Yes	27 (59%)	87 (56%)	
**Preoperative pathology**			0.415
CIN II	20 (43%)	72 (46%)	
CIN III	26 (57%)	84 (54%)	
**HR-HPV infection**			0.007
Negative	7 (15%)	121 (78%)	
Positive	39 (85%)	35 (22%)	
**Margin status**			0.012
Negative	8 (17%)	123 (79%)	
Positive	38 (83%)	33 (21%)	
LEEP depth (mm)	1.93 ± 0.40	1.88 ± 0.51	0.542
LEEP tissue size (cm^3^)	5.11 ± 1.48	5.07 ± 0.98	0.831
HGB (10^9^ g/L)	134 (125, 142)	136 (129, 142)	0.051
WBC (10^9^/L)	5.3 (4.5, 6.22)	5.36 (4.39, 6.37)	0.123
PLT (10^9^/L)	265.42 ± 66.11	273.79 ± 60.55	0.421
MPV (fL)	10.25 (9.6, 11)	10.3 (9.5, 11.2)	0.355
Neutrophil count (10^9^/L)	3.12 (2.61, 4.05)	3.08 (2.4, 3.83)	0.64
Lymphocyte count (10^9^/L)	1.77 ± 0.41	1.99 ± 0.64	0.029
PLR	196.6 ± 59.24	17.01 ± 49.56	0.003
NLR	2.72 ± 0.66	2.22 ± 0.69	<0.001

Measurement information conforming to normal distribution is expressed as mean ± SD. Qualitative information was expressed as *N* (%). When data did not conform to normal distribution, they were expressed as *M* (Q25 to Q75). Continuous variables were tested by *t*-test or Wilcoxon signed rank sum test. The chi-square test was used for categorical variables. *P* < 0.05. HR-HPV, high-risk human papillomavirus; CIN, cervical intraepithelial neoplasia; HGB, hemoglobin; WBC, white blood cell; PLT, platelet; MPV, mean platelet volume; NLR, neutrophil/lymphocyte ratio; PLR, platelet/lymphocyte ratio.

### 3.2 Predictive value of NLR for recurrence/residual in patients

**Figure 1:**
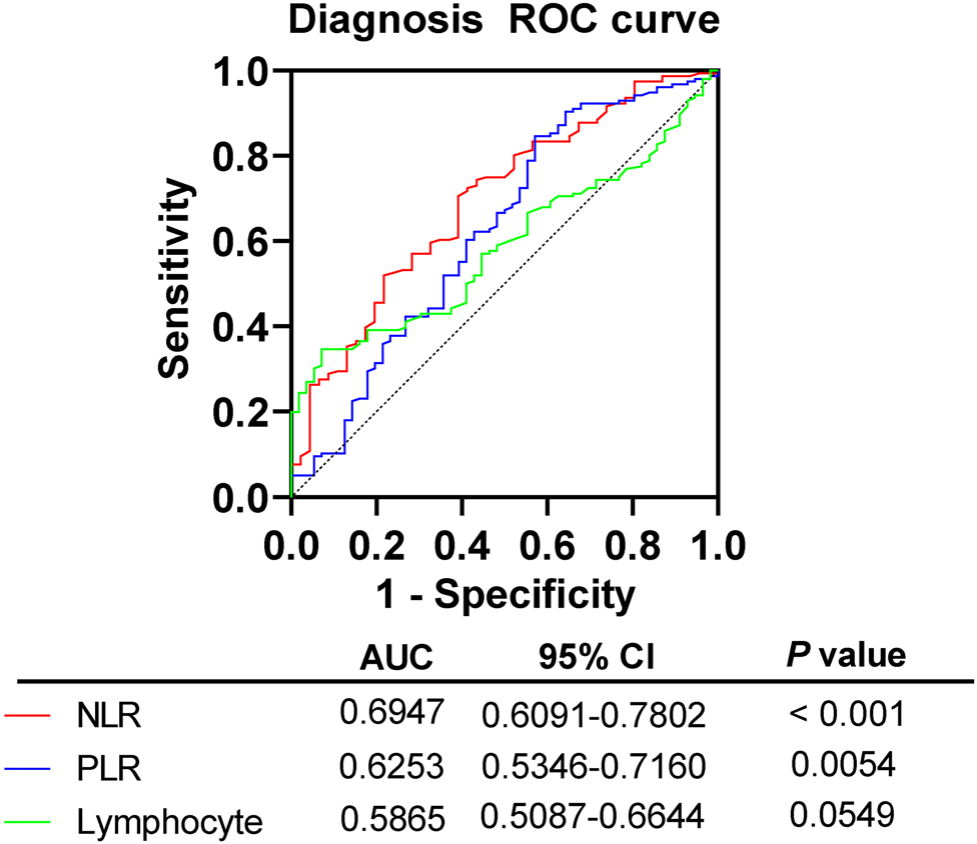
ROC curve assessing the predictive value of NLR for the occurrence of recurrence/residual in patients. *P* < 0.05 indicates significant difference.

With the recurrence/residual group as a positive sample, and the no recurrence/residual group as a negative sample, ROC curves were plotted. Table 2 and Figure 1 show that both NLR and PLR had good predictive efficacy. The AUC of NLR was 0.695 (95% CI 0.610–0.780, *P* < 0.05). When the cut-off value was taken as NLR > 2.655, the sensitivity of predicting the occurrence of recurrence/residual was 70.51% and the specificity was 60.87%. The AUC of PLR was 0.695 (95% CI 0.621–0.768, *P* < 0.05). When the cut-off value was taken as PLR > 209.6, the sensitivity of predicting the occurrence of recurrence/residual was 84.62% and the specificity was 42.86%. In addition, the AUC of lymphocytes was 0.587, which had no predictive validity for the occurrence of recurrence/residual in patients (*P* > 0.05).

**Table 2:** Predictive value of NLR for the occurrence of recurrence/residual in patients

Indices	Cut-off	Sensitivity (%)	Specificity (%)	*P* value
Lymphocyte count (10^9^/L)	2.28	35.26	92.86	0.055
PLR	209.6	84.62	42.86	0.005
NLR	2.65	70.51	60.87	<0.001

The value of NLR in predicting the occurrence of recurrence/residual in patients was assessed using the ROC curve. *P* < 0.05. NLR, neutrophil/lymphocyte ratio; PLR, platelet/lymphocyte ratio.

### 3.3 Kaplan Meier curve analysis of prognosis in patients with HSIL

**Figure 2:**
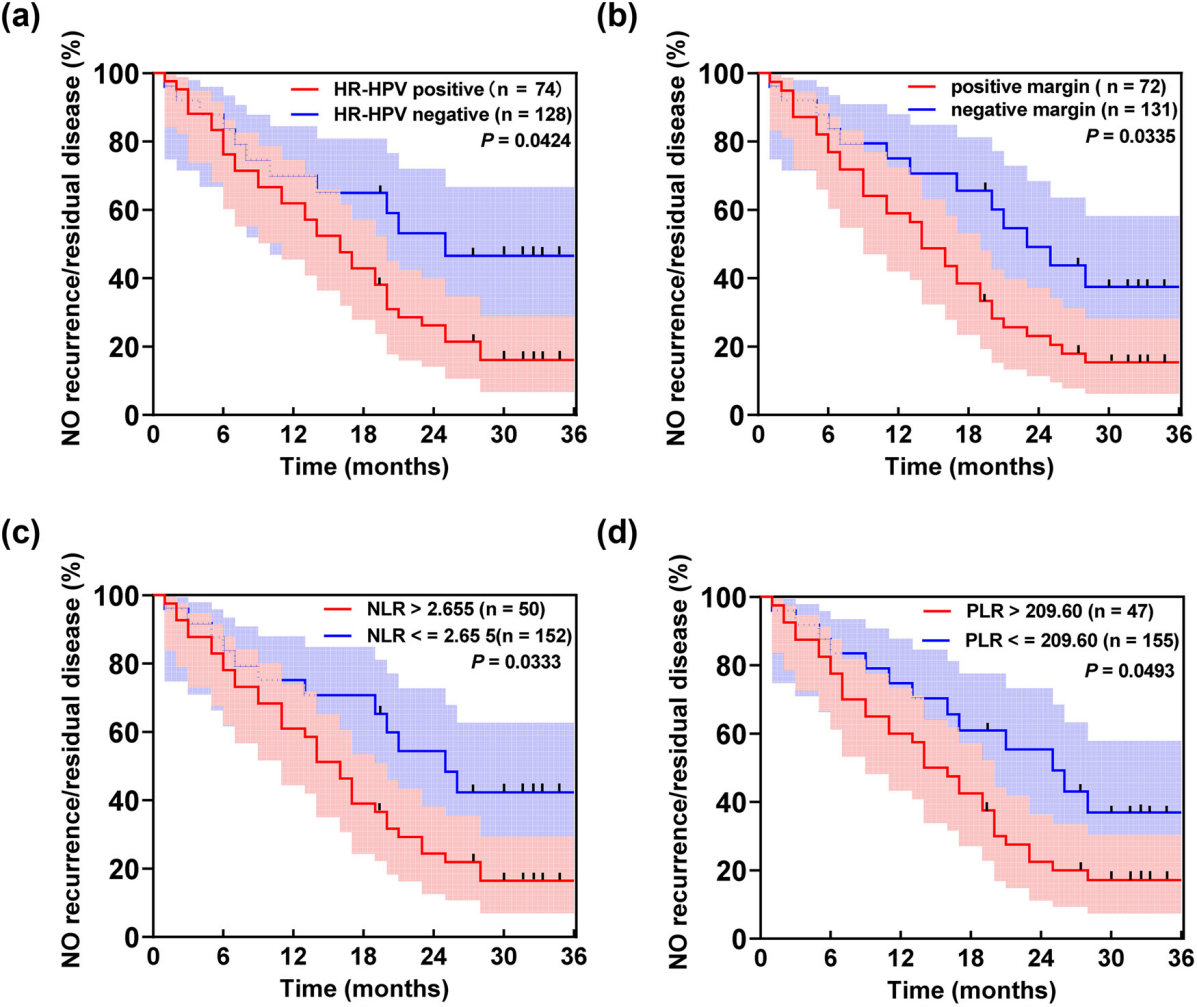
Kaplan Meier curves analyzing postoperative recurrence in HSIL patients. (a) HR-HPV infection promotes recurrence/residual at 36 months follow-up. (b) High PLR levels promote recurrence/residual at 36 months follow-up. (c) Positive margins promote recurrence/residual at 36 months follow-up. (d) High NLR levels promote recurrence/residual at 36 months follow-up.

From January 2017 to January 2022, a total of 209 HSIL patients were followed up to 202 cases, and 7 cases were lost to follow-up. Among them, the number of patients with recurrence or residual CIN II or CIN III was 46, and the number of patients with no recurrence or residual was 156, with a recurrence rate of 22.77%. Postoperative follow-up ranged from 1 to 3 years, with a mean follow-up of (22.48 ± 3.82) months and a median time to endpoint event of 16.5 months. Of the total 202 patients, groupings were made based on the HR-HPV, margin test results, and the optimal cut-off values of NLR and PLR to assess the incidence of recurrence/residual rate after surgery in these patients. The optimal cut-off values of NLR and PLR were 2.655 and 209.6, respectively, and patients were divided into groups with NLR ≤ 2.655 (*n* = 50) and NLR > 2.655 (*n* = 152), PLR ≤ 209.6 (*n* = 47) and PLR > 209.6 (*n* = 155). As shown in Figure 2, the postoperative incidence of recurrence/residual was higher in HSIL patients when HR-HPV positivity, positive margins, NLR > 2.655 (*n* = 50), and PLR > 209.6 (*n* = 47) were present, suggesting that the incidence of recurrence/residual after surgery was closely related to HR-HPV, margin condition, and levels of NLR and PLR.

### 3.4 Establishment of unifactorial and multifactorial Cox regression models to analyze risk factors for recurrence/residual in HSIL prognosis

**Figure 3:**
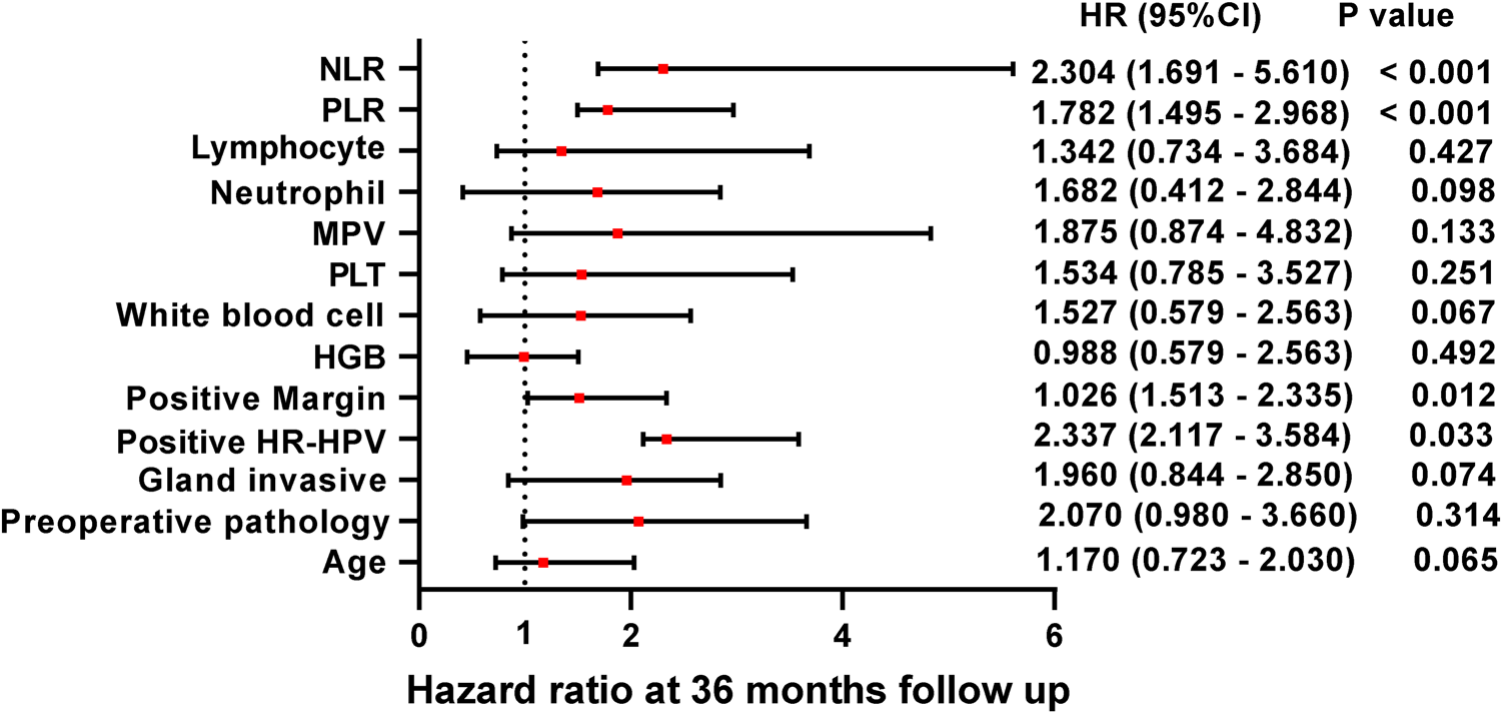
One-way Cox regression model analysis of possible risk factors for recurrence/residual after HSIL. *P* < 0.05 indicates significant difference.

**Figure 4:**
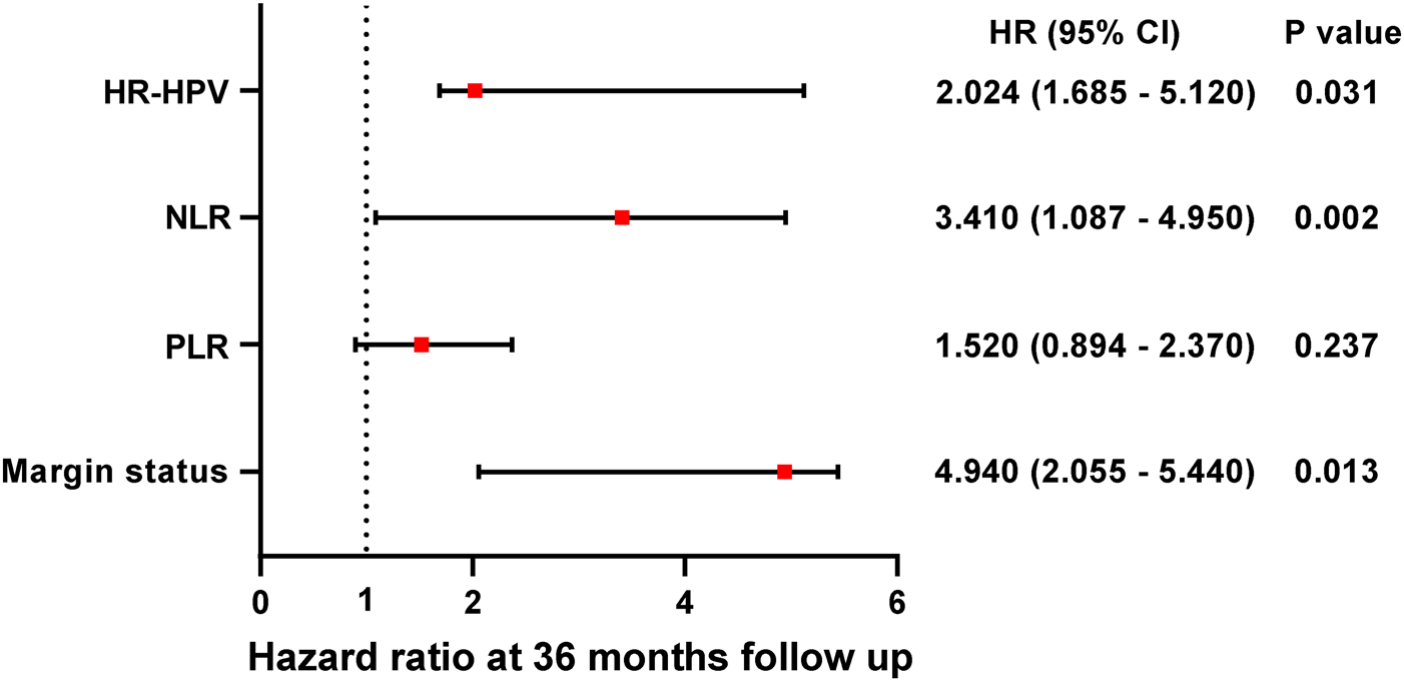
Multifactorial Cox regression modeling to analyze risk factors for recurrence/residual after HSIL. *P* < 0.05 indicates significant difference.

By univariate Cox analysis (Figure 3), patients with HR-HPV positivity, positive margins, and higher levels of PLR and NLR showed a significant cumulative risk during the follow-up period, with HRs of 2.337 (*P* = 0.033), 1.026 (*P* = 0.012), 0.782 (*P* < 0.001), and 2.304 (*P* < 0.001), respectively. Multifactorial Cox regression analysis showed that PLR had no effect on the prognosis of HSIL patients at follow-up. There was a difference in the cumulative risk of HR-HPV infection status (HR = 2.024, *P* = 0.031), positive margins (HR = 4.940, *P* = 0.013), and NLR (HR = 3.410, *P* = 0.002) (Figure 4). Among them, patients with positive margins and high NLR had a higher risk of developing recurrence or residual after surgery, which was 4.940 and 3.410 times higher than that of patients with negative margins and low NLR, respectively.

### 3.5 ROC curve analysis of the combined index to predict the occurrence of recurrence/residual in HSIL prognosis

**Figure 5:**
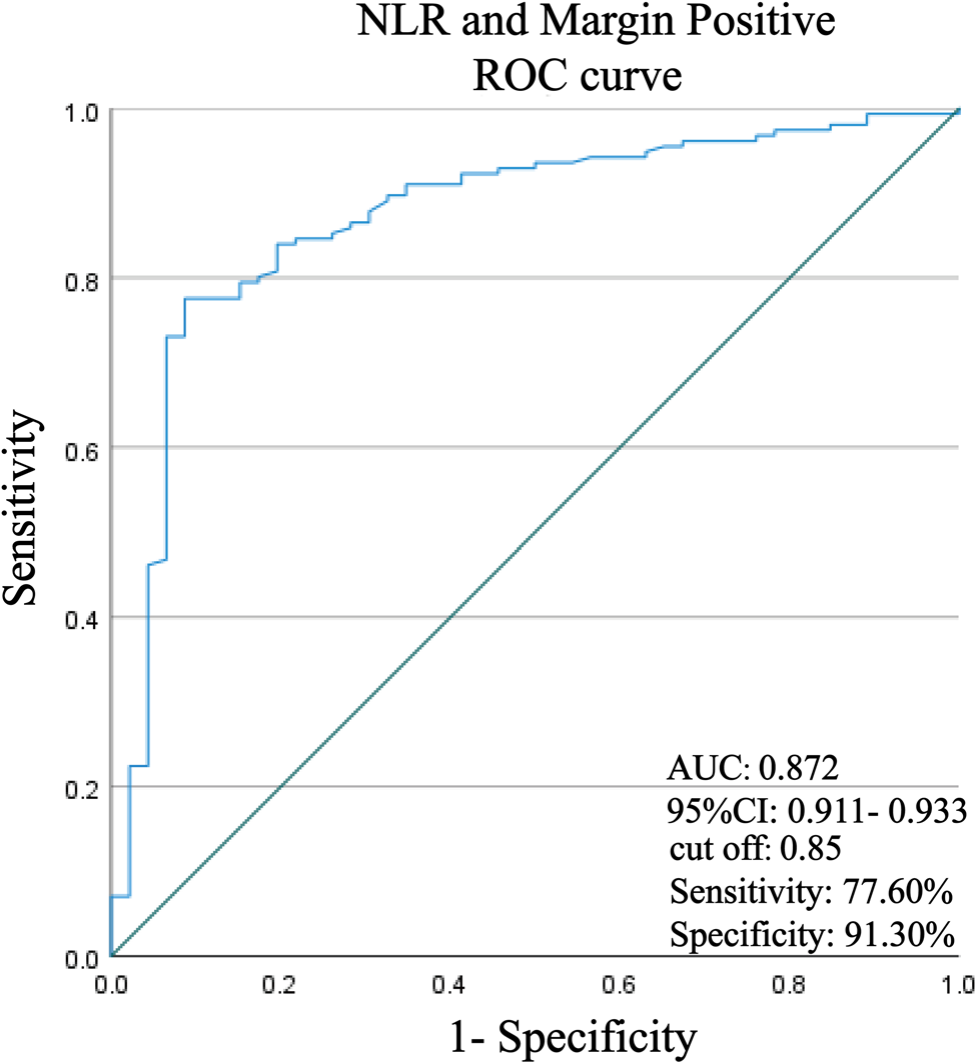
ROC curves analyzing the predictive efficacy of combined margin positivity and NLR on the prognostic occurrence of recurrence/residual in HSIL. *P* < 0.05 indicates significant difference.

A binary logistic regression analysis was performed with margin positivity and NLR as independent variables, resulting in a predictive probability value (combined indicator). ROC curve analysis was performed with the combined indicator as the new independent variable. The binary logistic regression analysis showed that the equation of the model was formulated as Logit (*P*) = 2.759 + 1.167 NLR + 2.872 Margin Positive (Table 3). Further results of the ROC curve showed that the AUC for margin positivity + NLR for predicting the occurrence of recurrence/residual for HSIL was 0.872 (95% CI: 0.911–0.933), with an optimal cut-off value of 0.85, a sensitivity of 77.60%, and a specificity of 91.30%. The predictive value of NLR combined with margin positivity for the prognostic occurrence of recurrence/residual in HSIL was significantly higher than the predictive efficacy of a single NLR index (Figure 5).

**Table 3:** Binary logistic regression with NLR and margin positive

Indices	*β*	SE	Wald	*P* value
NLR	1.167	0.348	11.233	0.001
Margin positive	2.872	0.457	39.547	0.000
Constant	2.759	0.895	9.504	0.002

NLR, neutrophil/lymphocyte ratio; SE, standard error.

## 4 Discussion

Cervical cancer is one of the most common malignant diseases in gynecology, and its progression from normal cervical changes to precancerous lesions such as HSIL and ultimately cervical cancer [16]. Chronic inflammation is considered one of the biological hallmarks of malignancy, releasing cytokines that induce angiogenesis and promote cancer development [17]. Neutrophils, platelets, and lymphocytes are indices that can be used as markers of inflammatory disease progression. Of these, NLR is commonly used as an indicator to assess disease severity and prognosis and to guide treatment [18]. However, to date, no study has confirmed the predictive value of NLR for HSIL, although it has been previously reported in patients with postoperative CIN. HPV testing combined with thin prep cytologic test remains the main strategy for postoperative treatment of cervical precancerous lesions. In this study, the correlation between NLR and margin condition and HSIL prognosis was described. As indicated, high NLR levels and positive margins were risk factors for prognosis of cervical lesions.

NLR is a systemic inflammatory indicator of the balance between anti-tumor immune response and pro-tumor inflammation and is an independent prognostic biomarker in various malignancies [19]. Neutrophil and lymphocyte counts are usually altered under inflammatory or infectious conditions. Neutrophils play a central role in inflammatory and infectious responses and are involved in inflammatory processes and immune cell killing functions. Lymphocytes are a component of the immune response and usually increase after infection [20]. It has been shown that an increase in NLR may indicate elevated levels of inflammation, decreased immune function, or stress responses in the body, while higher NLR values are usually associated with a poor prognosis in malignant tumors [21,22]. Our results showed that NLR levels were higher in the recurrence/residual group than in the no recurrence/residual group. Meanwhile, NLR has good efficacy in predicting the occurrence of recurrence or residual in HSIL patients after surgery, and high NLR level was a correlate affecting the occurrence of recurrence or residual in HSIL patients within 36 months after surgery. After establishing univariate and multivariate Cox regression models, it was shown that patients with high levels of NLR showed significant HR. NLR is an independent predictor of HSIL and its prognostic outcome [23]. We speculated that in persistent HR-HPV infection, chronic inflammation exists at the cervical site, leading to imbalance in the release of neutrophils, platelets, and lymphocytes, and disorganization of the body system [24]. At the same time, the repeated inflammatory response also damages vascular endothelial cells, further promoting platelet release and ultimately leading to elevated NLR levels. This study emphasizes the utility of NLR as a non-invasive biomarker in assessing disease outcomes and in guiding therapeutic decisions in a refined manner, which further strengthens the potential clinical value of NLR in risk stratification, therapeutic selection, and patient prognosis.

Positive margins after LEEP are a clear predictor of persistent/recurrent disease [25]. If the margins are negative, the probability of residual lesions is about 2–24%, while the probability of positive residual lesions can be as high as 30–60%. The risk of residual/recurrent lesions is as high as 5.3–17.7%, with positive pathologic margins strongly associated with cervical HSIL recurrence [26]. In this study, 38 patients with positive margins had recurrence or residual lesions, and 8 patients with negative margins had recurrence or residual lesions, suggesting that patients with positive margins have a higher probability of recurrence or residual lesions, and that negative margins may also have a poor prognostic outcome. Cox regression modeling analyzed that positive margins were a risk factor for patients with HSIL to develop recurrence or residual lesions after surgery. However, it has also been shown that positive margins do not imply treatment failure with LEEP. Simões and Campaner [27] and Lubrano et al. [28] found that 60–80% of patients with positive margins for HSIL showed regression during follow-up. Therefore, long-term follow-up and monitoring should be maintained after surgical treatment of HSIL. In this study, NLR combined with margin positivity was subjected to ROC curve analysis, and the results showed that the predictive value of NLR combined with margin positivity for the prognostic occurrence of recurrence/residual in HSIL was significantly higher than the predictive efficacy of individual NLR. However, existing studies have focused more on the value of NLR itself as a prognostic indicator rather than combined analysis with other clinicopathological features. Preoperative NLR is positively associated with the risk of positive surgical margin status in prostate cancer [29]. This suggests that NLR combined with positive margins is valuable for in-depth study to predict the prognostic occurrence of recurrence/residual in HSIL.

This study identified HR-HPV as an influencing factor in the occurrence of recurrent residual in HSIL patients after surgery. The results of a large number of epidemiologic and molecular biology studies have also demonstrated that high-risk HPV is a major contributor to cervical cancer [30,31]. The risk of residual/recurrent postoperative CIN2+ lesions was significantly higher in those with high-risk HPV-positive margins or uncertain margin status than in those with negative margins. However, high-risk HPV positivity is also an independent predictor of residual/recurrent CIN2+ postoperative lesions, with more than one-third of those with residual/recurrent lesions being high-risk HPV-negative early in follow-up [32,33], but the majority of them being HPV-positive in late routine follow-up [34]. It is further suggested that HPV infection is closely associated with the development of precancerous lesions and is a major factor in the progression of cervical precancerous lesions to cervical cancer. HPV positivity increases with the severity of the lesions, confirming the association of HPV infection with the development of cervical precancerous lesions and cervical cancer. In addition, HPV infection increases the risk of inflammatory lesions in the lower genital tract, and cervical deterioration increases with persistent HPV infection [35]. With the deepening of microecology research, several studies have found that cervical HR-HPV infection is associated with microecological dysfunction of lower genital tract flora [36,37]. This shows that early HPV prevention is essential for future tumor prevention. In clinical practice, HPV vaccination is one of the key measures in the cervical cancer prevention and control strategy. Currently, HPV vaccines available in the market include bivalent, quadrivalent, and nine-valent vaccines, all of which are effective in preventing more than 70% of cervical cancer occurrences, as well as other diseases caused by HPV.

The study had other limitations. Confounding factors such as smoking, alcohol consumption, or region were not included in the final analysis, which may have had an impact on the study’s data statistics. Therefore, additional multicenter studies and prognostic prediction models are needed for further research. The follow-up time span of some subjects was less than 3 years, and in the future we will use different regression models and correlation analyses for calibration to obtain more accurate results. In addition, the complexity of the tumor microenvironment and the multifactorial nature of cervical lesions require comprehensive analyses and longitudinal evaluations to determine the true predictive and prognostic utility of margin conditions and NLR in clinical practice.

In conclusion, our study suggests that high NLR levels and positive margins may be markers for predicting recurrent/residual lesions in HSIL after LEEP. Clinically, the combination of NLR level and margin condition testing may provide a new assessment method for predicting and managing recurrent/residual lesions in patients with HSIL after LEEP treatment. Future studies should include more cases and combine multiple metrics to analyze the diagnostic efficacy of cervical lesions and explore their mechanisms of action.
